# Optimizing Stroke Pathways: An Observational Audit of Door-to-CT Time, Thrombolysis, and Clinical Outcomes

**DOI:** 10.7759/cureus.97118

**Published:** 2025-11-17

**Authors:** Zobia Farrukh, Muhammad Umer Younas, Abdullah Yousaf, Sabih Nofal, Zarghoona Kamal, Burhan Anjum

**Affiliations:** 1 Internal Medicine, Fatima Jinnah Medical University, Lahore, PAK; 2 Internal Medicine, Aziz Fatimah Hospital, Faisalabad, PAK; 3 Internal Medicine, Chughtai Medical Centre, Gujranwala, PAK; 4 Vascular Surgery, Shalamar Hospital, Lahore, PAK; 5 Internal Medicine, Sir Ganga Ram Hospital, Lahore, PAK; 6 Internal Medicine, Akhtar Saeed Trust Hospital, Lahore, PAK

**Keywords:** door-to-ct time, intracerebral hemorrhage, recombinant tissue plasminogen activator, stroke, thrombolysis

## Abstract

Background

Stroke is a leading cause of disability and mortality worldwide, where timely intervention is crucial for favorable outcomes. This study aimed to evaluate door-to-CT time performance, eligibility for intravenous thrombolysis, and associated clinical outcomes in patients with acute stroke.

Methodology

This prospective observational study was conducted at Shalamar Hospital, Lahore, Pakistan, from February 2023 to February 2025. A total of 310 consecutive patients presenting with acute stroke symptoms were enrolled. Door-to-CT time, thrombolysis eligibility, and treatment details were recorded. Clinical outcomes were assessed at discharge using the modified Rankin scale (mRS).

Results

A total of 310 patients with clinically suspected stroke were enrolled in the study. The mean age of the patients was 61.4 ± 12.6 years, with 182 (58.7%) being male. Hypertension (225, 72.6%) and diabetes mellitus (138, 44.5%) were the most frequent comorbidities. A total of 201 (64.8%) patients underwent CT scanning within 25 minutes of arrival (door-to-CT time). Thrombolysis eligibility was established in 118 (38.1%) patients, of whom 96 (31.0% of the cohort) received intravenous recombinant tissue plasminogen activator (rt-PA). The mean door-to-needle time was 52.7 ± 14.6 minutes, with 71 (74%) of the treated patients achieving the <60-minute benchmark. Favorable outcomes (mRS score = 0-2) were observed in 50 (52.1%) thrombolysed patients compared to 71 (33.2%) non-thrombolysed patients (p < 0.01). Symptomatic intracerebral hemorrhage occurred in 6 (6.2%) treated patients, while overall in-hospital mortality was 34 (11.0%).

Conclusions

Achieving timely door-to-CT imaging significantly increases the likelihood of thrombolysis eligibility, while intravenous rt-PA improves functional outcomes with acceptable safety.

## Introduction

Stroke is a global health emergency and a leading contributor to long-term disability and premature death. The World Health Organization approximates that about 15 million people experience stroke a year, with one-third losing their lives and the other third left permanently disabled [1]. Due to restricted access to timely diagnostic and therapeutic care, the burden is very high, especially in low and middle-income countries. In high-income countries, life-saving opportunities have increased with the development of emergency systems and protocol-based management that has markedly increased survival and functional outcome [2]. The existence of these disparities demonstrates the importance of the uniformity, efficiency, and applicability of the method of stroke management. The commonly accepted guiding philosophy behind stroke management is that time is brain. Each minute without treatment after a stroke, the brain is deprived of approximately 14 billion synapses and 12 km of myelinated fibers, which supports the colossal consequences of untimely treatment [3]. This has led health systems to implement stringent time-associated quality metrics, including the most notable one to date, the time taken between the door and CT scan, the door-to-CT time. The American Heart Association and the European Stroke Association guidelines stipulate that non-contrast CT, the first imaging modality of choice, should be completed within 20 to 25 minutes of arrival in the hospital [4]. Imaging is very important, as it distinguishes ischemic stroke (where thrombolysis or thrombectomy can be assistive) from hemorrhagic stroke, where it is contraindicated. In resource-starved settings, even slight improvements in door-to-CT time have been found to be associated with considerable patient outcomes [5].

After neuroimaging, it is important to ascertain eligibility for reperfusion therapy, most importantly intravenous thrombolysis with recombinant tissue plasminogen activator (rt-PA). Originally recommended to be used up to three hours after the onset of symptoms, findings of large-scale trials have led to extending this time limit to 4.5 hours in well-specified patients [6]. Strict inclusion and exclusion criteria are applied to reduce the chances of complications, especially intracerebral bleeding. Some of the main factors to consider are age, any neurological deficit on baseline, blood pressure control, lack of recent surgery, gastrointestinal bleeding, and coagulopathy [7]. In addition, imaging results should not show hemorrhage or large established infarctions because they enhance the hazard of hemorrhagic transformation with treatment. In addition to the traditional window of treatment, other advanced modalities, such as CT perfusion and diffusion-weighted MRI, have allowed patient selection by individualizing treatment by the paradigm of salvageable penumbra [8]. These imaging modalities enable physicians to have greater confidence in determining patients who could still benefit from thrombolysis or thrombectomy outside of the standard therapeutic window, especially in large-vessel occlusions. This strategy is a paradigm shift away from rigid time-based standards to tissue-based decision-making, moving in line with the concept of precision-based medicine [9].

Despite the extensive evidence on the value of early intervention, there are still factors that hinder it in the real-world environment. Delays are usually experienced in the prehospital identification of stroke symptoms, transfer to the relevant facilities, and in-facility processes [10]. Media campaigns provide greater awareness of the problem (BE FAST: Balance, Eyes, Face, Arm, Speech, Time) and the early approach to the emergency department, as well as the creation of stroke-ready hospitals and regional stroke networks that facilitate the organization of referrals [11]. At the hospital level, the appointment of stroke units, emergency referral protocols through the emergency medical services, and the parallel processing process, whereby registration, history taking, and imaging requests are made at the same time, have also helped in eliminating the treatment delay. Tele-stroke services have also increased access to professional assessment in underserved locations [12].

Study objective

This study aimed to evaluate door-to-CT time performance, eligibility for intravenous thrombolysis, and associated clinical outcomes in patients with acute stroke.

## Materials and methods

Study design

This prospective observational study was conducted at Shalamar Hospital, Lahore, Pakistan, from February 2023 to February 2025. A total of 310 consecutive patients presenting with clinical features suggestive of acute stroke were enrolled. The sample size of 310 patients was determined to ensure adequate statistical power for evaluating the key outcomes of interest, namely, door-to-CT time compliance and thrombolysis eligibility based on the institutional stroke protocol aligned with the American Heart Association/American Stroke Association (AHA/ASA) guidelines [13].

Inclusion and exclusion criteria

Patients aged 18 years and above presenting with an acute neurological deficit within 24 hours of symptom onset and with clinical suspicion of stroke confirmed by neuroimaging were included. Exclusion criteria comprised patients with stroke mimics such as seizure, hypoglycemia, or migraine; those with incomplete records or who were lost to follow-up; and those who refused consent for participation.

Data collection

Upon arrival at the emergency department, all patients underwent immediate triage and neurological assessment using the National Institutes of Health Stroke Scale (NIHSS). The NIHSS is a standardized tool used to quantify neurological impairment in acute stroke. It evaluates 11 domains, including level of consciousness, gaze, visual fields, motor strength, ataxia, sensory function, language, speech, and neglect, with a total score ranging from 0 to 42. Scores were assigned by trained emergency physicians or neurology residents. For interpretation, a score of 0 indicates no stroke symptoms, 1-4 minor stroke, 5-15 moderate stroke, 16-20 moderate-to-severe stroke, and 21-42 severe stroke.

The time from hospital arrival to completion of the initial non-contrast CT scan was recorded as the door-to-CT time. Demographic variables, medical history, vascular risk factors, and prehospital delays were documented through structured proformas. Eligibility for intravenous thrombolysis was assessed according to international guidelines, which incorporated factors such as time since symptom onset, CT findings, and contraindications to thrombolysis. Eligible patients were administered rt-PA according to standard protocols, and treatment details, including door-to-needle time, complications, and early clinical outcomes, were recorded.

Clinical outcomes were assessed at discharge using the modified Rankin scale (mRS), a widely used measure of functional status after stroke [14]. The mRS ranges from 0 to 6, where 0 indicates no symptoms, 1 no significant disability, 2 slight disability, 3 moderate disability, 4 moderately severe disability, 5 severe disability (bedridden, requiring constant care), and 6 death. For analysis, scores of 0-2 were considered favorable outcomes (functional independence), while scores of 3-6 were categorized as poor outcomes.

The primary outcomes included the proportion of patients achieving the recommended door-to-CT time of less than 25 minutes, the percentage of patients deemed eligible for thrombolysis, and the proportion who received treatment. Secondary outcomes included in-hospital complications related to treatment and clinical status at discharge based on the mRS.

Statistical analysis

All collected data were entered into and analyzed using SPSS version 26.0 (IBM Corp., Armonk, NY, USA). Continuous variables such as age and time intervals were expressed as mean ± standard deviation, while categorical variables, including gender, risk factors, and treatment eligibility, were summarized as frequencies and percentages. The chi-square test was applied for comparisons between categorical variables, and the independent t-test was used for continuous data. A p-value of less than 0.05 was considered statistically significant.

## Results

A total of 310 patients with clinically suspected stroke were enrolled in the study. Of these, 182 (58.7%) were male, and 128 (41.3%) were female, with a mean age of 61.4 ± 12.6 years. Hypertension (72.6%) and diabetes mellitus (44.5%) were the most common comorbidities, followed by dyslipidemia (28.4%) and ischemic heart disease (19.0%). The majority of patients (84.2%) presented within six hours of symptom onset, whereas only 15.8% arrived after this window. The mean NIHSS score on admission was 13.2 ± 6.1, indicating moderate-to-severe neurological deficit in most patients (Table 1).

**Table 1:** Baseline demographic and clinical characteristics (n = 310). Data are presented as mean ± standard deviation for continuous variables and N (%) for categorical variables. NIHSS = National Institutes of Health Stroke Scale

Variable	Category/Definition	Mean ± SD/n (%)
Age	—	61.4 ± 12.6 years
Gender	Male	182 (58.7)
	Female	128 (41.3)
Comorbidities	Hypertension	225 (72.6)
	Diabetes mellitus	138 (44.5)
	Dyslipidemia	88 (28.4)
	Ischemic heart disease	59 (19.0)
Neurological severity	NIHSS on admission	13.2 ± 6.1
Presentation window	≤6 hours from symptom onset	261 (84.2)
	>6 hours from symptom onset	49 (15.8)

**Figure 1:**
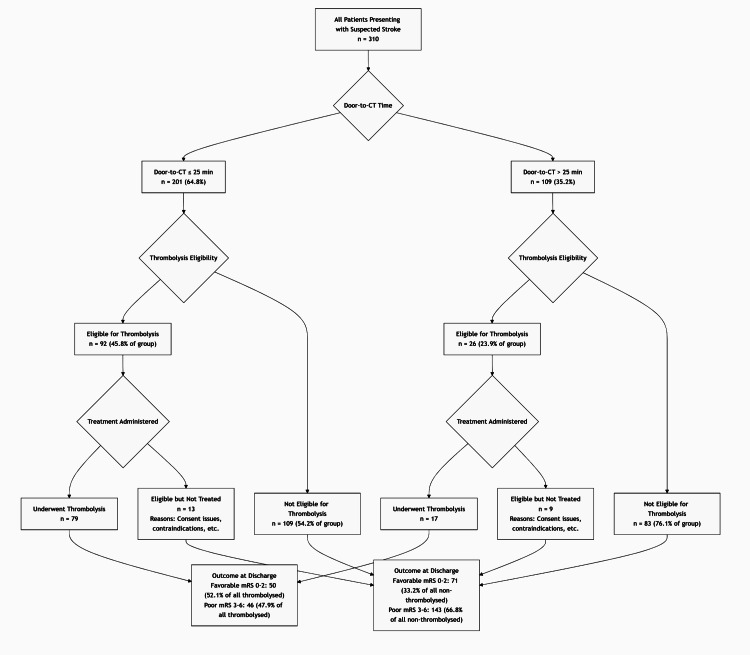
Patient flow and outcomes stratified by door-to-CT time and thrombolysis management. Flowchart of 310 acute stroke patients showing door-to-CT time, thrombolysis eligibility, and discharge outcomes. The ≤25-minute benchmark was achieved in 201 (64.8%) patients, with higher thrombolysis eligibility (45.8%) than those >25 minutes (23.9%, p < 0.01). Of the 118 eligible patients, 96 received thrombolysis. Treated patients had more favorable outcomes (mRS score = 0–2: 52.1% vs. 33.2%, p < 0.01). Common reasons for non-treatment included delayed consent, financial issues, and emerging contraindications. mRS = modified Rankin scale

The door-to-CT time met the ≤25-minute target in 201 (64.8%) patients, while 109 (35.2%) exceeded this threshold; the overall mean door-to-CT time across the cohort was 27.4 ± 11.2 minutes. Thrombolysis eligibility was established in 118 (38.1%) patients and excluded in 192 (61.9%), mainly due to delayed presentation beyond the therapeutic window (≤4.5 hours), contraindications such as intracranial hemorrhage, recent surgery, or uncontrolled hypertension. Intravenous rt-PA was administered to 96 (31.0%) patients and withheld in 214 (69.0%); the most common reasons for withholding were delayed arrival, hemorrhagic transformation on imaging, or medical contraindications identified on assessment. Among the thrombolysed group (n = 96), 71 (74.0%) achieved door-to-needle in <60 minutes, and 25 (26.0%) in ≥60 minutes; the mean door-to-needle time in treated patients was 52.7 ± 14.6 minutes (Table 2). The flow of patients through the study, based on door-to-CT time and thrombolysis management, is summarized in Figure 1.

**Table 2:** Stroke workflow timelines and thrombolysis metrics (n = 310). Data are presented as mean ± SD and n (%). *: Mean door-to-CT time for the overall cohort. ^†^: Percentages and mean door-to-needle time were calculated among thrombolysed patients only (n = 96). ^‡^: Also equals 18.6% of eligible patients (22/118). IV rt-PA = intravenous recombinant tissue plasminogen activator

Metric	Category/Definition	n (%)	Mean ± SD (minutes)
Door-to-CT time	≤25 minutes	201 (64.8)	—
	>25 minutes	109 (35.2)	27.4 ± 11.2*
Thrombolysis eligibility	Eligible for IV rt-PA	118 (38.1)	—
	Not eligible	192 (61.9)	—
Thrombolysis utilization	Thrombolysed	96 (31.0)	—
	Not thrombolysed	214 (69.0)	—
Door-to-needle time (treated only)	<60 minutes	71 (74.0)^†^	52.7 ± 14.6^†^
	≥60 minutes	25 (26.0)^†^	—
Eligible but not treated	—	22 (7.1)^‡^	—

Overall, 121 (39.0%) patients achieved a favorable functional status (mRS score = 0-2), while 189 (61.0%) had poor outcomes (mRS score = 3-6). In-hospital mortality occurred in 34 (11.0%) patients. Among those who received thrombolysis (n = 96), symptomatic intracerebral hemorrhage was documented in six (6.2% of treated) patients (Table 3).

**Table 3:** Clinical outcomes at discharge (n = 310). mRS = modified Rankin scale; ICH = intracerebral hemorrhage

Outcome	Frequency (n)	Percentage (%)
Favorable outcome (mRS 0–2)	121	39.0
Poor outcome (mRS 3–6)	189	61.0
In-hospital mortality	34	11.0
Symptomatic ICH after thrombolysis (n = 96)	6	6.2 (of treated)

Eligibility for thrombolysis was significantly more frequent when door-to-CT was ≤25 minutes: 92/201 (45.8%) were eligible versus 26/109 (23.9%) when door-to-CT exceeded 25 minutes (χ² p < 0.01). Conversely, non-eligibility was 109/201 (54.2%) in the ≤25-minute group and 83/109 (76.1%) in the >25-minute group, reinforcing the impact of rapid imaging on treatment candidacy (Table 4).

**Table 4:** Association between door-to-CT time and thrombolysis eligibility (n = 310). *: The chi-square test was used.

Door-to-CT time	Eligible for thrombolysis, n (%)	Not eligible, n (%)	Chi-square (χ²)	P-value
≤ 25 minutes (n = 201)	92 (45.8)	109 (54.2)	χ² = 12.11	<0.01*
> 25 minutes (n = 109)	26 (23.9)	83 (76.1)		

Thrombolysed patients (n = 96) had higher rates of favorable outcomes than non-thrombolysed patients (50/96, 52.1% vs. 71/214, 33.2%; χ² p < 0.01) and correspondingly fewer poor outcomes (46/96, 47.9% vs. 143/214, 66.8%). In-hospital mortality did not differ significantly (8/96, 8.3% vs. 26/214, 12.1%; p = 0.28). Symptomatic intracerebral hemorrhage, defined according to the AHA/ASA 2019 guidelines as any parenchymal hemorrhage temporally related to clinical deterioration (≥4-point increase in NIHSS score) within 36 hours of thrombolysis, occurred in 6/96 (6.2%) among treated patients and was not observed in the non-thrombolysed group (Table 5).

**Table 5:** Comparison of clinical outcomes between thrombolysed and non-thrombolysed patients (n = 310). *: The chi-square test was used. mRS = modified Rankin scale; ICH = intracerebral hemorrhage

Outcome	Thrombolysed (n = 96)	Non-thrombolysed (n = 214)	Chi-square (χ²)	P-value
Favorable (mRS 0–2)	50 (52.1)	71 (33.2)	χ² = 10.15	<0.01*
Poor (mRS 3–6)	46 (47.9)	143 (66.8)	χ² = 10.15	<0.01*
Symptomatic ICH	6 (6.2)	—	—	—
In-hospital mortality	8 (8.3)	26 (12.1)	χ² = 1.17	0.28

Delayed imaging (>25 minutes; n = 109) was more frequently observed among patients with higher baseline stroke severity and during periods of emergency department crowding. Specifically, NIHSS ≥15 was present in 56/109 (51.4%) with delayed imaging versus 69/201 (34.3%) with timely imaging (χ² p < 0.01), and arrival during peak emergency room hours occurred in 53/109 (48.6%) versus 62/201 (30.8%) (χ² p < 0.01). No significant differences were seen for male sex (60/109, 55.0% vs. 122/201, 60.7%; p = 0.34), age ≥65 years (57/109, 52.3% vs. 88/201, 43.8%; p = 0.14), hypertension (84/109, 77.1% vs. 141/201, 70.1%; p = 0.19), or diabetes mellitus (51/109, 46.8% vs. 87/201, 43.3%; p = 0.58) (Table 6).

**Table 6:** Factors associated with delayed door-to-CT time (>25 minutes) (n = 310). *: Statistically significant. The chi-square test was used. NIHSS = National Institutes of Health Stroke Scale; ER = emergency room

Variable	≤25 minutes (n=201)	>25 minutes (n=109)	Test statistic	P-value
Male sex	122 (60.7)	60 (55.0)	χ² = 0.91	0.34
Age ≥65 years	88 (43.8)	57 (52.3)	χ² = 2.18	0.14
Hypertension	141 (70.1)	84 (77.1)	χ² = 1.70	0.19
Diabetes mellitus	87 (43.3)	51 (46.8)	χ² = 0.31	0.58
NIHSS ≥15 on admission	69 (34.3)	56 (51.4)	χ² = 8.10	<0.01*
Arrival during peak ER hours	62 (30.8)	53 (48.6)	χ² = 9.44	<0.01*

There were no significant differences in mean age or admission NIHSS scores between thrombolysed and non-thrombolysed patients (Table 7). However, door-to-CT time was significantly shorter in patients scanned within 25 minutes compared to those with longer imaging delays (t = -21.3, p < 0.01) (Table 8). Similarly, patients with favorable outcomes had a shorter mean door-to-needle time than those with poor outcomes (t = -2.38, p = 0.02) (Table 9).

**Table 7:** Comparison of age and NIHSS on admission. The independent t-test was used. No statistically significant differences were observed. NIHSS = National Institutes of Health Stroke Scale

Variable	Thrombolysed (mean ± SD)	Non-thrombolysed (mean ± SD)	t-statistic (df)	P-value
Age (years)	59.8 ± 11.9	62.0 ± 12.9	–1.47 (308)	0.14
NIHSS on admission	12.4 ± 5.8	13.6 ± 6.2	–1.55 (308)	0.12

**Table 8:** Comparison of door-to-CT time. The independent t-test was used. Statistically significant at p < 0.05.

Variable	≤25 minutes (mean ± SD)	>25 minutes (mean ± SD)	t-statistic (df)	P-value
Door-to-CT time (minutes)	19.6 ± 3.8	39.5 ± 9.7	–21.3 (308)	<0.01

**Table 9:** Comparison of door-to-needle time and functional outcome (thrombolysed patients). The independent t-test was used. Statistically significant at p < 0.05.

Variable	Favorable outcome (mean ± SD)	Poor outcome (mean ± SD)	t-statistic (df)	P-value
Door-to-needle time (minutes)	50.1 ± 12.7	55.8 ± 15.9	–2.38 (94)	0.02

## Discussion

This study assessed the performance of stroke management protocols in a tertiary care hospital, focusing on critical time metrics and eligibility for reperfusion therapy. Among the 310 patients evaluated, nearly two-thirds achieved the recommended door-to-CT time of 25 minutes or less, and approximately one-third were deemed eligible for intravenous thrombolysis. The median time to CT was 27.4 minutes, and 64.8% of all patients met the guideline-recommended target. This is quite close to rates reported by developed stroke networks in which door-to-CT time averages of 20-30 minutes are the standard target. Nevertheless, delays continued to be experienced by over one-third of patients, especially at peak emergency room scheduling, and patients with severe neurological deficits in need of stabilization [15]. Identification of workflow bottlenecks, patient load, and insufficient staff availability have already been described as aspects that may contribute to imaging delays in previous literature. These results support the necessity of organizing the perfect logistics in the hospital, pre-notifications, and parallel processing, which will allow reducing the time of the imaging process to a minimum.

Regarding the use of reperfusion therapy, 38.1% of patients were considered eligible candidates for thrombolysis, whereas only 31.0% of the total population received treatment [16]. This gap, which is not as broad as that experienced in several low- and middle-income economies, denotes obstacles to access, namely, concurring late consent or contraindications and monetary limitations. In high-income settings, the rates of the usage of thrombolysis are low, making up no more than 10-15% of all ischemic strokes, mainly as a result of late presentation. The higher eligibility proportion in our study is probably due to the inclusion of patients with symptoms that occur within 24 hours and standardized assessment with objective criteria [17]. However, the fact that almost one-fifth of the population suitable to receive thrombolysis did not receive the latter allows solving the problem of patient counseling and the healthcare system to support all the more space. Notably, patients who underwent thrombolysis were significantly more functional at discharge, with 52.1% attaining functional independence (mRS score = 2) as opposed to 33.2% in the non-thrombolysed group. These findings are in line with history-making studies such as NINDS and ECASS III, which confirmed the effectiveness of the use of intravenous rt-PA in improving functional outcome [18]. Our low rate of symptomatic intracerebral hemorrhage (6.2%) was consistent with international observations and suggests that systematic use of inclusion criteria enhances the safety of hemostaseal treatment. The second important finding was that door-to-CT was well associated with thrombolysis eligibility. The likelihood that those who had the scan within 25 minutes could be eligible for reperfusion therapy nearly doubled that of the patients with delayed scans [19]. This observation confirms rapid imaging as a key to treatment, in line with international standards that place major emphasis on the so-called door-to-CT as a key indicator of care related to acute stroke. Imaging delays also limit the number of potential patients to receive thrombolysis and result in the loss of therapeutic potency delivered by thrombolysis, as cerebral ischemia is time-sensitive [20]. Variables that are likely to result in delay of imaging in our study were high NIHSS scores and arrival at peak emergency department hours. These findings concur with previous research, which indicated that patients in a critical condition, in need of airway stabilization, cardiac observation, or laboratory testing, often need to be stabilized before being transported to the CT unit. Although this is clinically necessary, emphasis on streamlined workflows and dedicated stroke pathways will be needed to balance stabilization/detection versus timely imaging [21,22].

This study has several limitations. First, this is a single-center study, likely making the results less generalizable because they are not based on a larger, multi-center sample. Second, our focus was only on in-hospital outcomes, and it was not possible to determine longer-term functional recovery beyond discharge. Third, the motivations behind eligible patients not receiving treatment were multifaceted and require more qualitative analysis to develop strategies that will overcome the barriers. Fourth, more advanced imaging techniques (e.g., CT perfusion and/or magnetic resonance) were not commonly accessible, which may have led to under-representation of patients who could have benefited from extended-window interventions. Fifth, we did not assess pre-stroke frailty or frailty indices as a covariate, although growing evidence indicates that frailty is an independent predictor of worse functional outcomes and mortality in acute stroke populations.

## Conclusions

Adherence to standardized stroke management protocols is associated with improved care quality in acute ischemic stroke. In this cohort, achieving the door-to-CT benchmark was linked to greater thrombolysis eligibility, and timely thrombolysis correlated with better functional outcomes at discharge. These descriptive findings highlight the importance of minimizing imaging delays and maintaining protocol adherence to optimize stroke management.
